# From Pluripotent Stem Cells to Organoids and Bioprinting: Recent Advances in Dental Epithelium and Ameloblast Models to Study Tooth Biology and Regeneration

**DOI:** 10.1007/s12015-024-10702-w

**Published:** 2024-03-18

**Authors:** Florian Hermans, Steffie Hasevoets, Hugo Vankelecom, Annelies Bronckaers, Ivo Lambrichts

**Affiliations:** 1 Department of Cardiology and Organ Systems (COS), Biomedical Research Institute (BIOMED), Faculty of Medicine and Life Sciences, Hasselt University, Diepenbeek, 3590 Belgium; 2 Laboratory of Tissue Plasticity in Health and Disease, Cluster of Stem Cell and Developmental Biology, Department of Development and Regeneration, KU Leuven, Leuven, 3000 Belgium

**Keywords:** Tooth Development, Stem Cells, Ameloblasts, Organoids, Amelogenesis, 3D Bioprinting

## Abstract

Ameloblasts are the specialized dental epithelial cell type responsible for enamel formation. Following completion of enamel development in humans, ameloblasts are lost and biological repair or regeneration of enamel is not possible. In the past, in vitro models to study dental epithelium and ameloblast biology were limited to freshly isolated primary cells or immortalized cell lines, both with limited translational potential. In recent years, large strides have been made with the development of induced pluripotent stem cell and organoid models of this essential dental lineage – both enabling modeling of human dental epithelium. Upon induction with several different signaling factors (such as transforming growth factor and bone morphogenetic proteins) these models display elevated expression of ameloblast markers and enamel matrix proteins. The advent of 3D bioprinting, and its potential combination with these advanced cellular tools, is poised to revolutionize the field – and its potential for tissue engineering, regenerative and personalized medicine. As the advancements in these technologies are rapidly evolving, we evaluate the current state-of-the-art regarding in vitro cell culture models of dental epithelium and ameloblast lineage with a particular focus toward their applicability for translational tissue engineering and regenerative/personalized medicine.

**Graphical Abstract:**

**Future perspectives for in vitro modeling of dental epithelium and ameloblasts.** Development of iPSC and organoid models that can reliably generate dental epithelium and ameloblast-like cells, together with advances in 3D bioprinting, provide promising tools for enamel research. Advanced models will provide new avenues for development of enamel repair/regeneration approaches, for testing of dental materials or drugs, studying host-pathogen and/or cell-cell interactions, in vitro modeling of enamel diseases (e.g. amelogenesis imperfecta) and developing novel insights in fundamental tooth biology (e.g. regulation of amelogenesis, lineage specification). Abbreviations: iPSC: induced pluripotent stem cells; TO: tooth organoids; DE: dental epithelium; AB: ameloblast.

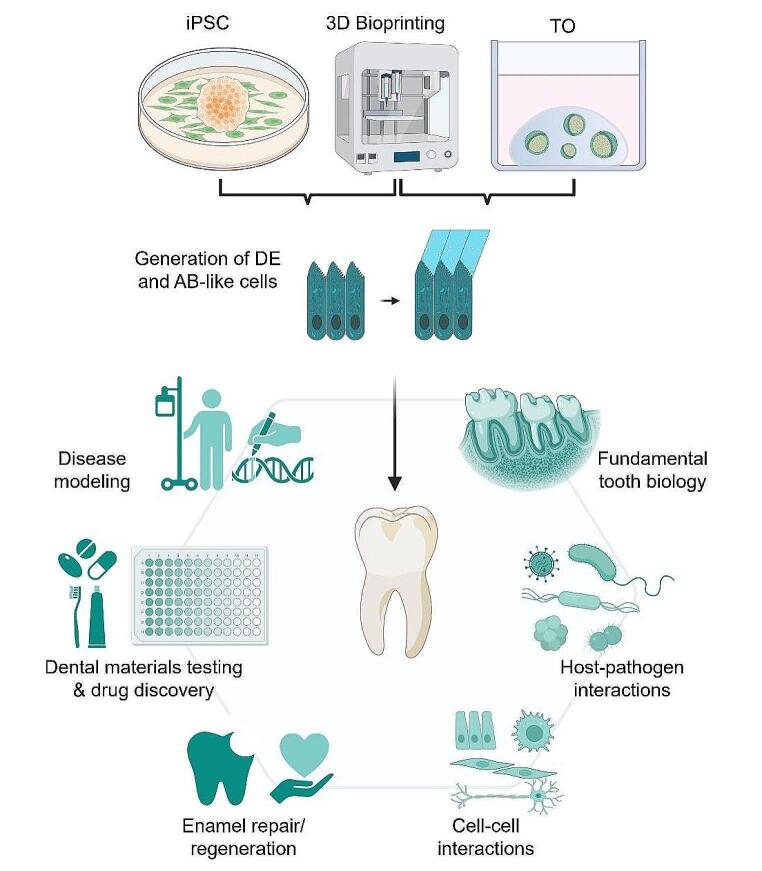

## Background

Reciprocal epithelial-mesenchymal interactions are the driving forces throughout the various stages of tooth development. Whereas the dental mesenchymal compartment contributes to most cells in mature tooth, including dental pulp and periodontal ligament, as well as to the dentin layer which is produced by odontoblasts, the dental epithelium eventually differentiates into ameloblasts (AB), the specialized cell type that forms enamel covering the crown of the tooth [1, 2].

**Fig. 1:**
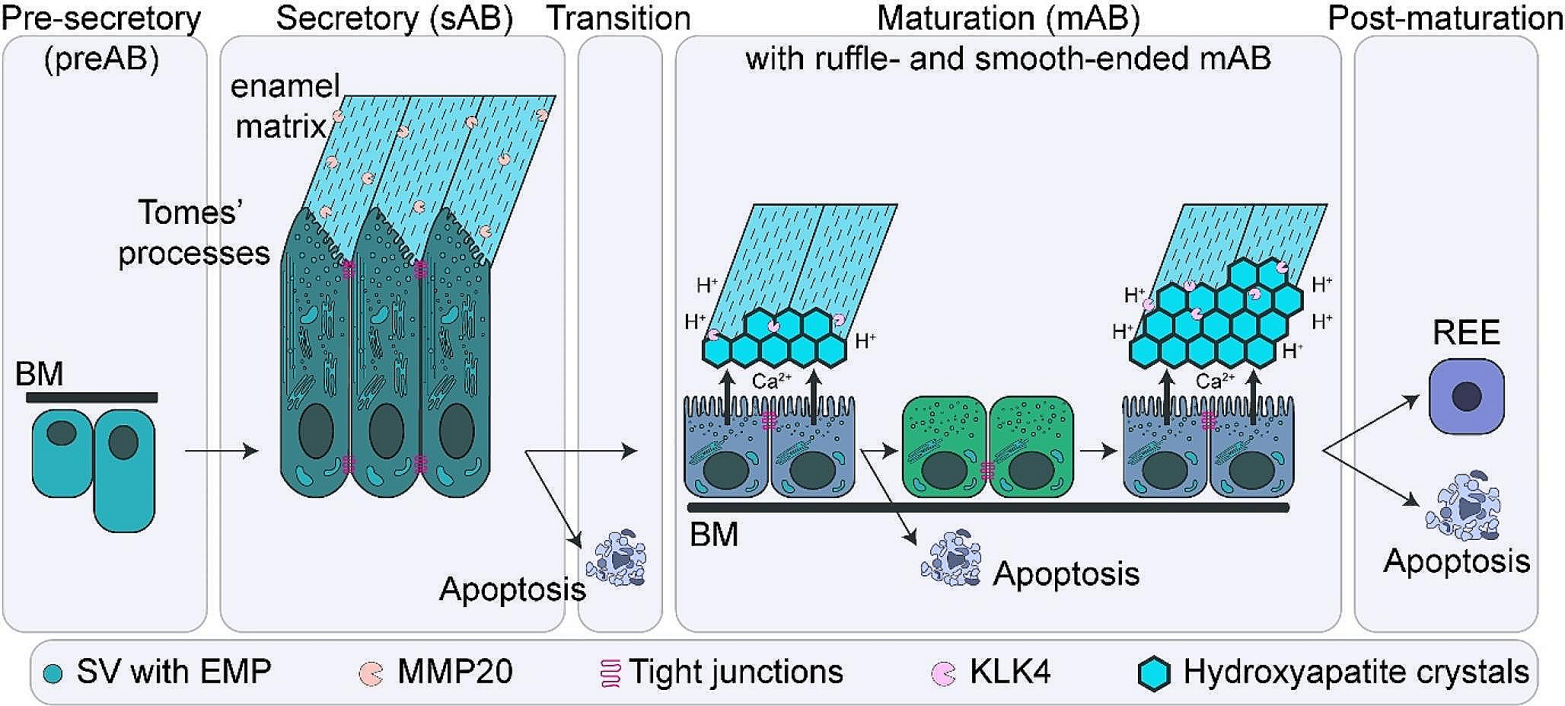
Schematic overview of the ameloblast lifecycle. Ameloblasts (AB) undergo a linear differentiation trajectory during amelogenesis consisting of four main stages, as defined by histological and functional properties: (1) pre-secretory stage, (2) secretory stage, (3) transitional stage and (4) maturation stage. Upon completion of amelogenesis (“post-maturation”), most AB undergo apoptosis, or contribute to reduced enamel epithelium (REE), causing the tooth to lose all enamel-reparative/regenerative capacity. Abbreviations: preAB: presecretory ameloblasts; sAB: secretory-stage ameloblasts; mAB: maturation-stage ameloblasts; BM: basement membrane; Ca^2+^: calcium; H^+^: hydrogen ion SV: secretory vesicles; EMP: enamel matrix proteins (e.g. AMELX, AMBN); MMP20: matrix metalloproteinase 20; KLK: kallikrein-related kinase 4; REE: reduced enamel epithelium

The ameloblast lifecycle consists of four well-defined morphological and functional stages (Fig. 1). In the first step, inner enamel epithelium cells (presecretory ameloblasts; preAB) initiate cytodifferentiation by inducing differentiation of adjacent dental mesenchyme into odontoblasts, which deposit a fine layer of pre-dentin at the future dentinoenamel junction, in turn reciprocally instructing preAB to differentiate into secretory-stage AB (sAB) [3, 4]. During this process, preAB break through the basement membrane at the dentinoenamel junction, elongate from short cuboidal into tall columnar cells, and form so-called Tomes’ processes containing the cells’ secretory machinery at their apical enamel-forming ends. sAB secrete enamel matrix proteins such as amelogenin (AMELX), enamelin (ENAM) and ameloblastin (AMBN) to form a soft protein-rich enamel matrix, which is modified and stabilized by secreted proteases such as matrix metalloproteinase 20 (MMP20) [5, 6]. Using a variety of mineral and bicarbonate transporters, AB can drive mineral growth within the matrix, with each AB eventually forming a thin enamel rod or enamel crystallite [7]. In the third stage, AB transition from the secretory to the maturation stage by retracting their Tomes’ processes and depositing a new basal lamina. During this phase, and again during the final stage, approximately 25% of AB undergo apoptosis [8, 9]. Eventually, in the fourth stadium, maturation-stage AB (mAB) cycle between two morphologies at the enamel surface: ruffle-ended and smooth-ended, and enamel matrix protein expression shifts from predominantly AMELX and AMBN to the expression of odontogenic, AB associated (ODAM) and amelotin (AMTN) [10]. mAB degrade and reabsorb the enamel protein matrix through secretion of proteolytic enzymes (e.g. kallikrein-related peptidase-4 (KLK4)), while the enamel crystallites continue to grow and expand. Importantly, pH fluctuation has been found essential to the maturation phase, and pH levels appear closely intertwined with mAB morphology and function [11–13]. Acidification, which gradually increases during the ruffle-ended phase, is essential for proper maturation of hydroxyapatite crystallites. Once completed, the initially proteinaceous enamel matrix is highly mineralized with only little amounts of protein remaining. At the end of their lifecycle, all AB either contribute to the reduced enamel epithelium (a thin epithelial layer covering the enamel prior to eruption) or undergo apoptosis. After this phase, enamel is considered largely unable to be repaired or (re-)generated.

**Fig. 2:**
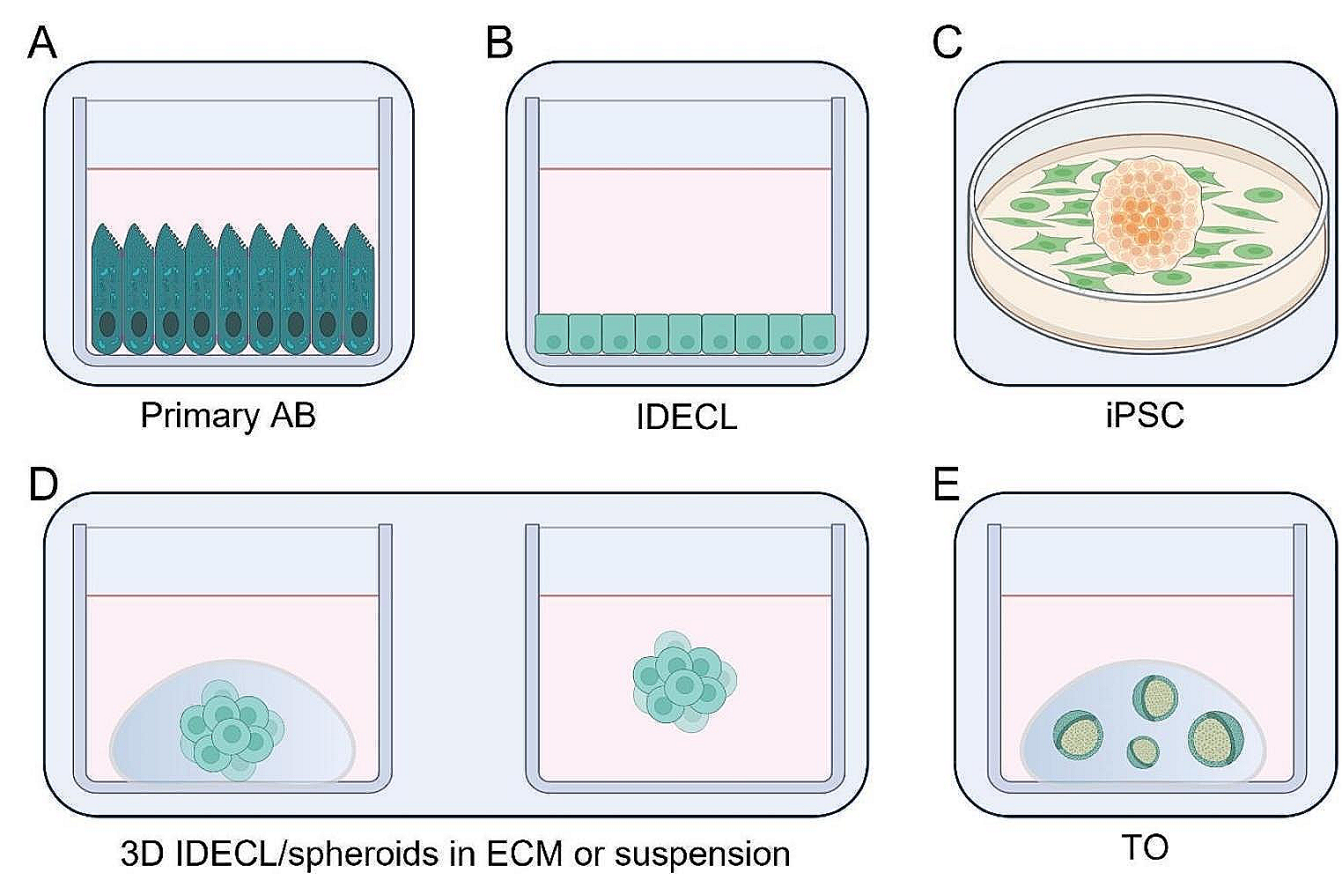
Overview of in vitro models of dental epithelium and ameloblasts. Firstly, primary AB **(A**) and IDECL **(B)** were the gold standard to study DE and AB in vitro. Recently, iPSC models **(C)** and 3D culture, either embedded in ECM (**D**, left), in suspension (**D**, right) or as organoids **(E)**, have revolutionized the field. Abbreviations: AB: ameloblasts; IDECL: immortalized dental epithelial cell lines; iPSC: induced pluripotent stem cells; ECM: extracellular matrix; TO: tooth organoids

Each compartment may be separately affected in patients, as is the case in congenital disorders of tooth enamel and dentin (i.e. amelogenesis and dentinogenesis imperfecta, respectively; or as the enamel lesions caused by caries) [14, 15]. Alternatively, both dental mesenchyme- and epithelium-derived compartments may be jointly affected as for example in deep caries (which passes through enamel and dentin layers toward the pulp chamber) or in case of tooth loss or agenesis. To repair tooth defects in the field of restorative dentistry and tissue engineering, the strategy must therefore depend on the tooth compartment affected. Thus, to develop biological tooth repair and replacement strategies, reliable methods are required to first expand dental mesenchymal and epithelial compartments, and subsequently obtain differentiated dentin-producing odontoblasts and enamel-forming AB, respectively. Whereas considerable research efforts are aimed at developing tooth bioengineering and regenerative strategies, most studies start from the dental mesenchymal compartment (focusing on dental pulp and periodontal ligament) which alone will not be able to reconstitute the dental epithelial compartment and allow enamel regeneration [16]. Therefore, in this review, we evaluate the current state-of-the-art regarding in vitro cell culture models of dental epithelium, and their potential for expansion and AB production (Fig. 2; Table 1).

**Table 1:** Overview of dental epithelium/ameloblast models

Model	Expandability	Genomic stability	Patient-derived	AB-like differentiation	Dental origin/epigenetic memory	Tooth type specific features	References
Primary AB	no	yes	no	N/A	yes	undetermined	[17–23, 30]
IDECL	yes	no	no	yes	yes	undetermined	[31–38, 40, 41, 43, 46]
iPSC	yes, prior to differentiation	yes	yes	yes	no	undetermined	[36, 42, 48–53, 112–115]
3D IDECL	no	no	no	yes	yes	undetermined	[40, 62–64, 116]
3D spheroids	yes	yes	no	no	yes	undetermined	[66]
Ex vivo tooth germ culture	no	yes	no	yes	yes	yes/undetermined	[67–78]
3D iPSC	yes, prior to differentiation	yes	yes	yes	no	undetermined	[52, 53]
3D human TO	yes	yes	yes	yes	yes	undetermined	[87, 117]
3D mouse TO	yes	yes	no	yes	yes	yes	[88]

## Main text

### Primary Ameloblast Cells

Primary AB from rodent molars and incisors can be harvested and used for in vitro experimentation (Fig. 2A) [17–23]. Typically, mandibles are isolated, surrounding soft tissues are carefully excised, and the incisor enamel organ is micro-dissected. Using anatomical features, such as molar landmarks or morphological differences between stratum intermedium and papillary cells underlying respectively sAB or mAB, it is possible to delineate between secretory, transitional and maturation stages (for instance, see Houari et al. for video guidance on this procedure) [21, 24–26]. However, the use of spatial landmarks is not always straightforward, as positioning or tissue integrity might alter with age, or in pathological conditions [26]. Using enzymatic and mechanical digestion, distinct AB populations can be separately isolated and cultured in 2D, with serum-containing medium. A major drawback of this method is the possibility of contamination by other surrounding cells, such as stratum intermedium or connective tissue cells. Therefore, several studies have used fluorescent labeling to identify AB (e.g. using AMELX, AMBN) and/or exclude stromal cells (e.g. using CD90 as a marker) [18, 20]. Additionally, isolated AB must be used within 24-72 h of isolation, as these cells no longer proliferate and lack survival and expandability in vitro. Importantly, the same methodology can be employed to isolate AB fractions for RNA and protein analyses. Due to the difficulty maintaining primary AB in culture, many studies combine primary AB with other in vitro AB models (e.g. immortalized dental epithelial cell lines; see below).

Freshly isolated AB are most frequently used for in vitro study of the mechanisms and (dys-)regulation of ion metabolism (including ion channels and transporters, ameloblast-specific organelle biology) involved during enamel mineralization, for example using calcium imaging, (whole cell) patch clamping and ultrastructural imaging [18–21]. However, primary AB are employed in a wide array of study designs, for instance to evaluate the influence of environmental exposure (e.g. exposure to fluoride or bisphenol A and other endocrine disruptors) on different stages of amelogenesis, or to study developmental amelogenesis defects (e.g. in combination with mouse models of the developmental enamel disorder amelogenesis imperfecta) [22, 23, 27–29].

Although human primary dental epithelium/AB-like cell cultures starting from fetal tooth germ have been reported, their use is infrequent, and ethical concerns limit their use [30, 31]. Moreover, in contrast to primary AB from rodents, culture of fetal human AB-like cells was achieved by digestion and selective subculture of dental epithelial cells from fetal tooth germ, likely resulting in spontaneous immortalization (see further), and not all cells expressed AB-like features (e.g. expression of enamel matrix proteins). Therefore, primary rodent AB remain an essential in vitro tool for the tooth biologist to advance our understanding of fundamental AB and enamel biology. As new models are developed, their AB-like phenotype should be benchmarked against primary AB cells. Finally, due to their short-lived nature and the lack of human-derived primary AB, these cells are not usable for use in tissue engineering and regenerative medicine strategies.

### Immortalized Dental Epithelial Cell Lines

Secondly, numerous immortalized dental epithelial cell lines (IDECL; recently reviewed by Zeng et al. (2023)) have been developed from the enamel organ of various species including mouse (e.g. ALC, LS8 and EOE-2 M/3 M), rat (e.g. HAT-7, SF2), pig (PABSo-E) and human (h-ALC) (Fig. 2B; Table 2) [31–39]. Similarly, other human immortalized cell lines were also derived from primary ameloblastoma (a benign tumor of the odontogenic epithelium), Hertwig’s Epithelial Root Sheath (HERS, a transient dental epithelial population crucial for root development), or of Epithelial Cell Rests of Malassez (ERM; located in the dental follicle and/or periodontal ligament) [40–44]. In general, immortalization of odontogenic tissues has been achieved using spontaneous immortalization, overexpression of viral oncogenes (e.g. SV40 or HP16 E6/E7 genes) or ectopic expression of human telomerase reverse transcriptase (hTERT) [32]. Genomic manipulation to produce immortalized cell lines has been associated with increased genomic instability (i.e. a state in which genomic mutations occur at higher frequency), including chromosomal instability (defined as a cellular state during in which unwarranted chromosomal changes in number and structure occurring at a high rate) [32, 45]. As a consequence IDECL may become unstable and heterogeneous (within and between different laboratories), resulting in skewed and unreproducible results. A community effort to characterize the genomic profiles of IDECL, and implement standardized genomic monitoring (e.g. karyotyping, comparative genome hybridization), would be a tremendous step forward. Nonetheless, these models recapitulate some key features of AB (e.g. expression of enamel matrix proteins, *in vitro/vivo* mineralization potential) making them a frequently used tool to probe processes involved in AB differentiation and develop novel 3D cell culture models (see further). Due to their ease of culture, IDECL are typically used to benchmark newly developed in vitro AB models – although IDECL are less physiologically relevant than isolated primary AB.

**Table 2:** Overview and properties of commonly used immortalized dental epithelial cell lines (IDECL).

Cell line	Species	Tissue origin	Immortalization strategy	Developmental stage	Tooth type	AB phenotype	References
ALC	Mouse	Enamel organ	Spontaneous	Newborn	Molar (mandibular and maxillary)	mAB	[37, 46]
LS8	Mouse	Enamel organ	SV40	Newborn	Molar (mandibular)	sAB	[35, 46]
EOE-2 M/3 M	Mouse	Enamel organ	HPV16 E6/E7	Postnatal day 7	Molar (mandibular)	Unknown	[38]
HAT-7	Rat	Enamel organ	Spontaneous	Postnatal day 6	Incisor (mandibular)	Unknown	[34]
SF2, SF2-24	Rat	Enamel organ	Spontaneous	Postnatal day 6	Incisor (mandibular)	preAB	[36, 39]
PABSo-E	Pig	Enamel organ	SV40	Unerupted (unknown)	Molars	Unknown	[33]
h-ALC	Human	Enamel organ	Spontaneous	Fetal (21 weeks)	Pooled	Unknown	[31]
AM-1, HAM1	Human	Ameloblastoma	HPV16 E6/E7; hTERT	Various	Unknown, molar (HAM1)	Unknown	[43, 44]
Not named	Human	HERS, ERM	SV40; spontaneous	Postnatal (unknown)	Third molar	Unknown	[40, 41]

Interestingly, some IDECL have been reported to skew toward either preAB (SF2), sAB (LS8) or mAB (ALC) phenotype, which should be evaluated for other IDECL (Table 2) [46]. In addition, comparisons between different IDECL (e.g. to study molar- and incisor-specific differences in the dental epithelium) are challenging because most IDECL were developed using different methodologies, from different tooth types, at different developmental time points and from various species (Table 2). One could envision a collaborative effort to develop and make available IDECL using standardized methodologies and from corresponding developmental time points. In the meantime, researchers must exert caution and select the most appropriate IDECL for their study design. Thus, although IDECL can be expanded indefinitely (i.e. useful to obtain the required cell numbers for regenerative medicine strategies), presence of genomic instability and resulting genetic abnormalities further limits overall translatability and usefulness for regenerative medicine and tissue engineering endeavors.

### Induced Pluripotent Stem Cells

A third method to model dental epithelium and AB is based on differentiation from induced pluripotent stem cells (iPSC) (and to a lesser extent on embryonic stem cells (ESC)) (Fig. 2C). iPSC are obtained by reprogramming somatic cells to dedifferentiate and acquire a pluripotent stem cell phenotype capable of self-renewal and differentiation into cells of all three germ layers. Although several methods exist, reprogramming is usually achieved by forced overexpression of specific transcription factors (TF) such as *octamer-binding transcription factor 4* (*Oct4*), SRY (sex determining region Y)-box 2 (*Sox2)*, *Kruppel-like factor 4* (*Klf4*) and *c-myc*.

**Fig. 3:**
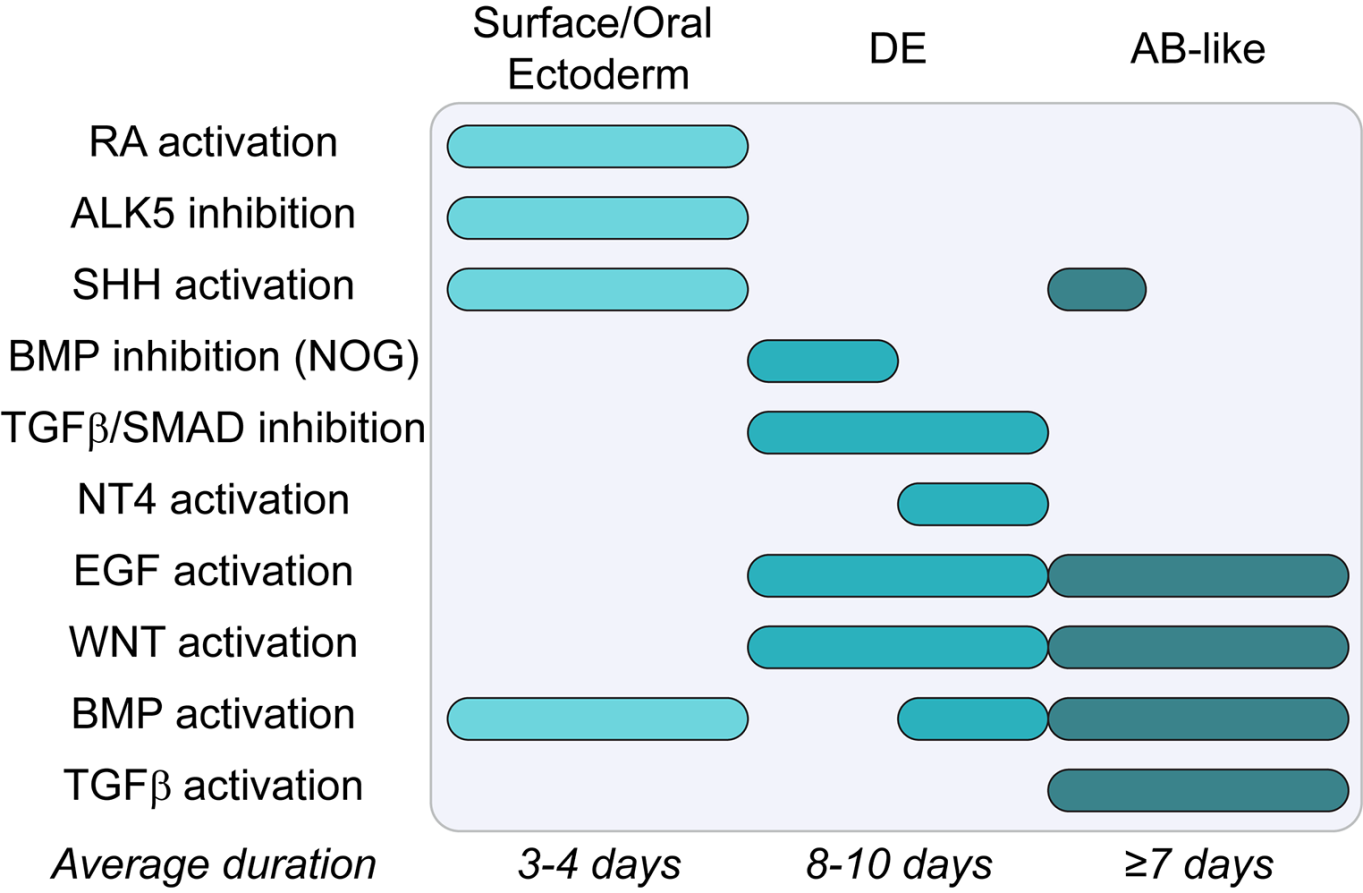
Overview of common signaling pathway modulation strategies for generation of ameloblast-like cells (based on Tables 3 and 4) in iPSC and tooth organoid models. Abbreviations: DE: dental epithelium; AB: ameloblast; RA: retinoic acid; ALK5: activin receptor-like kinase 5; SHH: sonic hedgehog; BMP: bone morphogenetic protein; NOG: Noggin; TGFβ: transforming growth factor β; SMAD: mothers against decapentaplegic; NT4: neurotrophin 4; EGF: epidermal growth factor; WNT: Wingless-type MMTV integration site

Overall, three fundamental approaches (either alone or in combination) have been applied to induce dental epithelium and/or AB differentiation from mouse and human iPSC. As a first strategy, through either co-culture with IDECL (as feeders instead of mouse embryonic fibroblasts) or application of conditioned medium from IDECL, iPSC are induced to acquire an epithelial phenotype and/or AB-like features (typically assessed by evaluation of enamel matrix protein expression) [36, 42, 47–49]. Alternatively, most recent protocols aim to replicate the sequential steps of AB development by mirroring key developmental cues (Fig. 3; Table 3) [50–53]. Typically, these procedures start by developing embryoid bodies from iPSC (i.e. spontaneously formed 3D aggregates comprising cells from all three germ layers upon suspension culture of iPSC), which are then guided toward an epithelial oral ectodermal fate, typically through stimulation of bone morphogenetic protein (BMP), retinoic acid (RA) and/or sonic hedgehog (SHH) signaling pathways. Next, dental epithelial fate is acquired, and cells can be pushed toward AB-like cells. Currently, no clear consensus protocol has been achieved, although modulation of BMP, SHH, Wingless-type MMTV integration site (WNT), epidermal growth factor (EGF) and transforming growth factor β (TGFβ) signaling appear crucial herein (Fig. 3; Table 3) [50–53]. Thirdly, through co-culture with dental mesenchymal cells (derived from embryonic day 14–16 molar tooth germs; see further), shown to possess tooth-inductive capabilities, and subsequent in vivo maturation (typically by transplantation under the kidney capsule), iPSC can differentiate into AB-like cells and produce mineralized tissues [48, 49, 53]. Although used as a protocol to induce differentiation in earlier studies, this methodology has shifted toward a validation tool, i.e. to confirm the in vivo differentiation potential of obtained differentiated cell products rather than the endpoint [50, 52, 53].

**Table 3:** Overview of recently developed protocols to differentiate iPSC toward dental epithelium and/or ameloblast-like cells

Study	iPSC	Surface/Oral Ectoderm	DE	AB-like differentiation
**Kim** (2021) [50]	**day 0–4** - Embryoid bodies formed with 10,000 cells/well	**day 4–8** - Fibronectin coating − 192 EBs/100mm^2^ - DMEM/F12 + 1X N2, BMP4 (25 ng/mL), RA (1 mM)	**day 8–12** KSFM^1^ + NOGGIN (100 ng/mL), EGF (20 ng/mL) **day 12–16** - Replace NOGGIN with BMP4 (25 ng/mL)	**In vivo transplantation**: Recombined with E14 molar dental mesenchyme and transplanted into kidney capsule (8 weeks)
**Miao** (2021) [115]	**day 0–2** - Embryoid body formation	**day 2–5** Supplement with SB43152 (5 µM)	**day 5–10** DMEM/F12 + EGF (20ng/mL), FGF2 (25 ng/mL), 1X B27, BMP4 (12.5 ng/mL), RA (1 µM), LiCl (20 mM)	**day 10–17** α-MEM + 10% serum, ascorbic acid (50 µg/mL), calcium chloride (2.0 mM), and β-glycerophosphate (10 mM).+ TGF-β1 (3 ng/mL) + EGF (10 ng/mL) + LiCl (15 mM)
**Kim** (2023) [52]	**day 0–4** - Embryoid bodies formed with 10,000 cells/well	**day 4–8** - Fibronectin coating − 192 embryoid bodies/100mm^2^ - DMEM/F12 + 1X N2, BMP4 (50 ng/mL), RA (0.1 mM)	**day 8–12** KSFM^1^ + NOGGIN (100 ng/mL), EGF (1 µg/mL) **day 12–26 (organoid culture)** Transfer to Matrigel (3D), with KBM^2^ + 50% WNT3A conditioned medium, 5% RSPO1 conditioned medium, EGF (100 ng/mL), FGF10 (100 ng/mL), dibenzazepine (10 µM), NOGGIN (200 ng/mL), 1X N2, 1X B-27, Nicotinamide (10 mM), A83-01 (0.5 µM), N-acetylcysteine (1 mM), Y-27,632 (10 µM)	**day 26–40** Replace NOGGIN with BMP4 (100 ng/mL) **In vivo transplantation**: Recombined with E14 molar dental mesenchyme and transplanted into kidney capsule (12 weeks)
**Alghadeer** (2023) [53]	Cultured on Matrigel-coated plates until confluent	**day 0–3** Switch to ‘ameloblast base media’ ^3^ + 0.1X S7 supplement + β-mercaptoethanol (0.1 µM) + SAG (400 µM))	**day 3–8** Supplement with BMP4 (150 pM) daily **day 8–12** Replace BMP4 with LDN-193,189 (1 µM), CHIR99021 (5 µM), EGF (500 pM), NT4 (3.5 µM)	**day 12–16** - Replace LDN-193,189 and NT4 with BMP4 (300 pM) and TGF-β1 (800 nM) **day 16-further** − 3**D suspension culture** in ‘ameloblast base medium’ + Y-27,632 (10 µM) - **Co-culture with DPSC**: 3D spheroids or monolayers - **In vivo transplantation**: either intramuscularly (8 weeks) or into kidney capsule (8 weeks)

^1^ KSFM: keratinocyte serum-free medium;

^2^ KBM: keratinocyte basal medium

^3^ Either EpiCult-C (Stem Cell Technologies #05630) or RPMI1640 (Thermo #11,875,093) mixed 1:1 with EpiLife (Thermo #MEPI500CA)

**Table 4:** Overview of recently developed protocols to culture tissue-derived epithelial tooth organoids, and achieve ameloblast-like differentiation

Study	Organoids	Expansion conditions	In vitro differentiation	In vivo differentiation
**Hemeryck** (2022) [87, 117]	Adult human tooth, developed from ERM in dental follicle	SFDM^1^ + 1X B27, 1X N2, L-glutamine (2 mM), N-acetylcysteine (1.25 mM), WNT3A (200 ng/mL), RSPO1 (200 ng/mL), NOGGIN (100 ng/mL), FGF10 (100 ng/mL), FGF8 (200 ng/mL), FGF2 (20 ng/mL), A83-01 (0.5 µM), nicotinamide (10 mM), IGF1 (100 ng/mL), cholera toxin (100 ng/mL), SHH (100 ng/mL)	**day 0–7**: culture in expansion medium **day 7–14**: switch to KSFM^2^ + calcium (0.09 mM), EGF (1 ng/mL), bovine pituitary extract (50 µg/mL)	**Subcutaneous transplantation**: dissociated organoid cells were seeded with Matrigel in hydroxyapatite scaffolds and transplanted subcutaneously for 4 weeks.
**Hermans** (2023) [88]	Postnatal (day 7) mouse molar and incisor tooth organoids	SFDM^1^ + 1X B27, 1X N2, L-glutamine (2 mM), N-acetylcysteine (1.25 mM), WNT3A (200 ng/mL), RSPO1 (200 ng/mL), NOGGIN (100 ng/mL), FGF10 (100 ng/mL), FGF8 (200 ng/mL), FGF2 (20 ng/mL), A83-01 (0.5 µM), nicotinamide (10 mM), IGF1 (100 ng/mL), cholera toxin (100 ng/mL), SHH (100 ng/mL), EGF (20 ng/mL)	**day 0–7**: culture in expansion medium **day 7–14**: switch to SFDM + 1X B27, BMP2 (100 ng/mL), BMP4 (50 ng/mL), EGF (20 ng/mL), FGF2 (20 ng/mL), TGF-β1 (4 ng/mL)	**Subcutaneous transplantation**: day 7 organoids were seeded with Matrigel in hydroxyapatite scaffolds and transplanted subcutaneously for 1 week. **Chorioallantoic membrane (CAM) assay (*in ovo*)**: day 7 organoids were seeded with Matrigel onto the CAM of fertilized chicken eggs (9 days) for 1 week.
**Kim** (2023) [89]	Adult (9 week) mouse incisor organoids	DMEM/F12 + 1X B27, 1X N2, N-acetylcysteine (1 mM), Y-27,632 (10 µM), 50% WNT3A conditioned medium, 5% RSPO1 conditioned medium, EGF (100 µg/mL), NOGGIN (200 µg/mL), FGF10 (100 µg/mL), dibenzazepine (10 mM), A83-01 (0.5 µM), nicotinamide (10 mM)	**day 0–14**: culture in expansion medium **day 14–28**: suspension culture in expansion medium supplemented with SAG (concentration not provided by authors)	**Kidney transplantation**: day 14 organoids were first cultured in suspension for 4 weeks and subsequently transplanted into the kidney capsule for 8 weeks.

**Underlined**: Shared factor in all expansion media

^**1**^ SFDM: serum-free defined medium, see Hemeryck or Hermans et al. for detailed composition [87, 88]; ^2^ KSFM: keratinocyte serum-free medium

Even though iPSC are a potent tool with numerous advantages, such as low invasiveness of procurement, recapitulation of patient-specific genetic background, limitless supply of cells, and reduced risks of immune rejection when using a patient’s own cells, several aspects may affect their functionality for enamel and/or tooth tissue engineering. Firstly, with the aim of pursuing regenerative medicine, there are inherent risks associated with the use of oncogenes for reprogramming and the genetically unstable nature of reprogramming, which may promote tumor formation [54, 55]. However, use of non-integrating vectors may mitigate some of these risks [55–57]. Secondly, donor-specific genetic and epigenetic background may have undesired effects on the final product. It is well established that after reprogramming, iPSC still retain some epigenetic memory of their tissue of origin. Depending on the application, the presence of epigenetic memory can either impair or enhance lineage-specific differentiation of iPSC. For example, reprogrammed pancreatic β-cells were able to more robustly differentiate into insulin-producing β-cells compared to fibroblast-derived iPSC [58]. Several research groups have established iPSC lines from dental mesenchymal origins, such as from the dental pulp or periodontal ligament, whereas dental epithelium-derived iPSC have not yet been established [59–61]. To our knowledge, the effects of epigenetic memory have not yet been evaluated for iPSC in dental applications. Further study is required to develop dental epithelium-derived iPSC- (e.g. from ERM found in dental follicle or periodontal ligament, as done for tooth organoids; see below), and scrutinize the effects of epigenetic memory and iPSC origin (e.g. skin fibroblast-, dental mesenchyme- or epithelium-derived) on dental epithelium/AB differentiation efficacy and potential.

### 3D Cell Models of the Dental Epithelium

#### Non-Expandable 3D Culture of Immortalized Dental Epithelial Cell Lines and Dental Spheroids

In parallel with the development of IDECL and AB differentiation protocols for iPSC, researchers have been turning toward 3D culture of these cells in an attempt to further improve differentiation toward AB-like cells (Fig. 2D). Most simply, IDECL (ALC, SF2 or HAT-7) were expanded in a traditional 2D cell culture setting before seeding the cells (often together with fetal or postnatal dental mesenchymal cells) in an extracellular matrix (ECM), typically Matrigel (i.e. an ECM produced by a mouse sarcoma cell line and rich in basement membrane proteins such as laminin, collagen IV and entactin) (Fig. 2D) [62, 63]. Compared to their 2D counterparts, 3D-cultured IDECL typically showed enhanced expression of enamel matrix proteins such as AMELX. However, it was not evaluated whether these models could be expanded once seeded in the 3D environment.

Tadaki et al. (2016) were able to improve AB-like differentiation of immortalized rat SF2 cells by 3D suspension culture in fabricated polydimethylsiloxane scaffolds [64]. However, this system did not show any long-term expandability of cells once cultured in 3D. Also in 3D suspension culture, Tsunematsu et al. (2016) reported the formation of spheroids from immortalized human ERM. Although formed ERM-spheroids displayed stemness features, no expandability or dental epithelium/AB-like differentiation was reported [40]. Similarly, Natsiou et al. (2017) established the 3D culture of primary incisor labial cervical loop cells in Matrigel, displaying a keratinized stratified squamous epithelial phenotype. This study did not report any expression of AB markers, nor the possibility to expand these cultures [65]. In contrast, several years earlier, Chang et al. (2013) described a procedure for the generation of long-term expandable mouse dental epithelial stem cell spheres also established from the incisor labial cervical loop [66]. Although 3D-cultured spheres lacked expression of AB markers such as enamel matrix proteins, some enamel matrix protein expression could be induced by dissociating the spheres and seeding the cells in 2D together with a defined differentiation cocktail containing mineralization-inducing factors typically applied to stromal cells, such as dexamethasone, β-glycerophosphate, ascorbic acid and elevated calcium concentration.

Therefore, similarly to other dental epithelium model systems, 3D-cultured IDECL and dental spheroids still present important shortcomings to be used as a tool for tooth and/or enamel bioengineering strategies. Clear consensus protocols have not emerged and only Chang et al. (2013) showed the development of a long-term expandable culture of primary dental epithelial cells from mouse incisor, albeit lacking in AB-like differentiation potential [66].

#### Ex Vivo Organ Culture of Tooth Germs

**Fig. 4:**
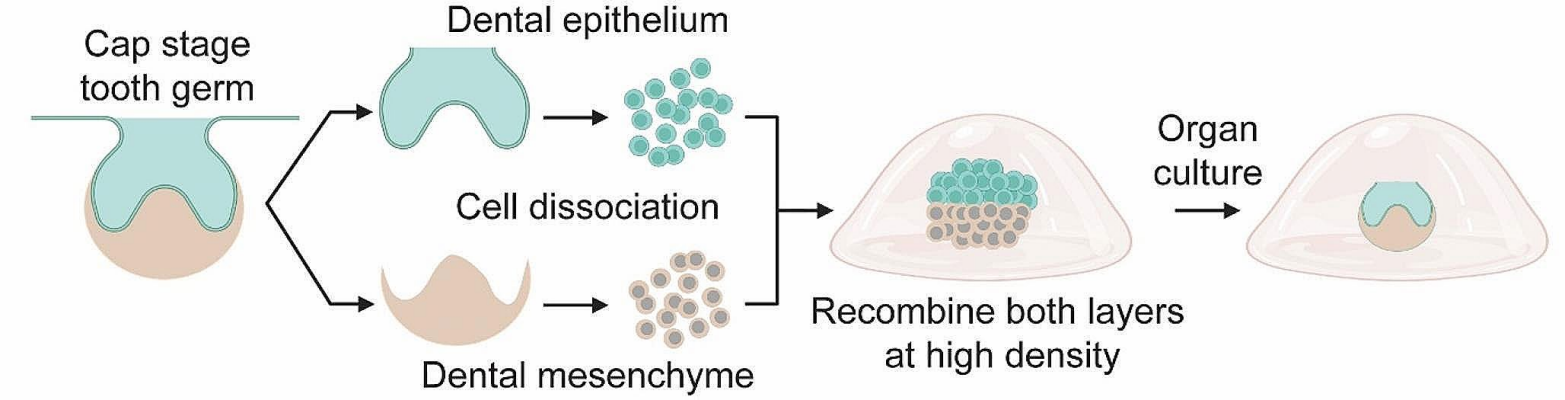
Schematic overview of ‘bioengineered tooth germ’ technology. After collection of tooth germs from embryonal (typically day 14–16) mouse molar and incisor tooth germs, dental epithelium and dental mesenchyme are separately dissociated into single cells, and subsequently recombined at high cellular density in collagen droplets for ex vivo organ culture. Typically, organ cultures are transplanted into the kidney capsule of mice after 2–7 days of ex vivo culture to drive cytodifferentiation and formation of tooth structures

Ex vivo organ culture of embryonic tooth germs, typically followed by in vivo (e.g. subrenal) transplantation of the formed structures, has been a foundational staple in the field since the 70s [67, 68]. Since the early 2000s, researchers have been working on developing biological whole tooth replacement strategies using tooth germ culture approaches. In 2002, Young. et al. (2017) recombined dissociated primary dental epithelial and mesenchymal cells obtained from porcine tooth germs in a degradable polyglycolate/poly-L-lactate and poly-L-lactate-co-glycolate scaffold, which successfully formed mature tooth structures (including enamel, dentin and pulp) after 20–30 weeks of transplantation in rat omentum [69]. Similar results were obtained when both cell types from postnatal day 4 rat molar tooth germs (cap stage) were seeded on polyglycolate/poly-L-lactate and poly-L-lactate-co-glycolate scaffolds and transplanted into the rat omentum or a tooth extraction site [70, 71]. These developments were further improved by development of ‘bioengineered tooth germ’ technology (Fig. 4). Starting from embryonic day 14.5 mouse molar and incisor tooth germs (cap stage), dental epithelium and dental mesenchyme are separately dissociated into single cells, and subsequently recombined in collagen droplets [72]. Essentially, at this developmental stage, the dental mesenchyme possess tooth-inductive capacity, enabling development of mature dental epithelial tissue when recombined with other non-dental epithelial cell sources (e.g. human gingival epithelium, keratinocytes, iPSC) [48–50, 52, 73, 74]. Importantly, both cell types must be seeded at high density and compartmentalized within adjacent layers allowing direct cell-cell contact to enable success. Following ten days in vitro growth, transplantation under the subrenal capsule or in a tooth extraction site, tooth structures can be formed. Since its conception, this technology has been further enhanced and validated: bioengineered tooth germs can generate fully functioning and mature tooth when transplanted into a tooth extraction site, even becoming innervated and vascularized, and containing periodontal ligament [75, 76]. Moreover, using a size-control device, the shape and length of the bioengineered tooth germ can be regulated so that it is similar in size to a natural tooth [76]. Interestingly, in one bioengineered tooth germ multiple tooth primordia are frequently observed [72]. By developing a ligature-based method to split these primordia into individual tooth germs the yield could be enhanced [77]. Further, bioengineered tooth germ technology has also successfully been applied in beagles from both embryonic and postnatal tooth germs: at postnatal day 30 the tooth germs of permanent premolars were at a suitable stage (i.e. cap stage) to generate bioengineered tooth germ [78]. Importantly, this study also demonstrated autologous transplantation of postnatal canine bioengineered tooth germ, bringing the bioengineered tooth germ technology closer to the clinic.

Unfortunately, application of bioengineered tooth germ technology is severely limited by the requirement of cap stage dental (mesenchymal) tissues, which are not easily collected from patients. Third molars or wisdom teeth are the last teeth to develop, are considered rudimentary and are routinely extracted, making them the most suitable candidate to collect tooth germs from. However, the average age of the initiation of third molar mineralization is between 7 and 10 years old, which would require tooth germs to be extracted before this point to develop bioengineered tooth germ [79–81]. Thus, the application of the bioengineered tooth germ technology for human biological tooth replacement requires further advances in obtaining postnatal dental stem cell sources, which can be guided toward a cap stage phenotype. Finally, as previously indicated, ex vivo recombination with murine tooth-inductive embryonal dental mesenchyme, and subsequent in vivo transplantation, are frequently used in conjunction with newly developed dental epithelium/AB models to validate their in vivo differentiation potential, which is essential for their further application in tooth bioengineering.

#### Tooth Organoids

In the last 15 years, the emergence of the organoid technology has drastically reshaped biomedical research by providing researchers the ability to mirror (parts of) organs and their development, phenotype and function in vitro [82–84]. Organoids are typically defined as self-forming and self-organizing 3D cell models that strongly mimic the physiology and sometimes morphology of their in vivo counterpart (i.e. much more than traditional 2D monolayer cell cultures). Establishment of organoids can start from iPSC/ESC, tissue-resident epithelial stem cells or stem cell-containing tissue fragments, and has been successful from many organs. In addition to their tissue epithelium-mirroring properties, organoids developed from epithelial tissue (stem) cells are highly and long-term expandable while genomically and transcriptionally stable, and retain their phenotypical and functional properties during the extensive culture time [83, 84]. Additionally, through optimization of the organoid medium (typically shifting from stemness-promoting medium components to known differentiation-inducing signals) or co-culture with other cell types, several organoid models may acquire a differentiated cell state [84]. In addition, organoid lines can be established from patients’ diseased tissue biopsies and mimic key aspects of the disease phenotype in vitro, making them powerful tools for disease modeling and for personalized medicine, either to determine the optimal therapeutic action or for use in regenerative medicine approaches [83–86]. The fact that organoids are highly and long-term expandable, genomically stable, as well as able to differentiate has strongly boosted the path to future regenerative therapies, for instance as recently shown for human bile ducts [85]. This landmark study showed for the first time the possibility to transplant human organoids into a live human organ (ex vivo), with organoids functionally engrafting in the host tissue. Thus, organoid technology is an exciting tool for many researchers strongly enabling both fundamental and applied research endeavors. Moreover, development of organoids from dental epithelial tissue would overcome many of the limitations associated with current options (Supplemental Appendix).

Recently, our group was the first to develop epithelial organoids starting from human and postnatal mouse tooth, followed by Kim et al. who derived incisor organoids from adult mouse (Fig. 2E; Table 4) [87–89]. Human tooth(-derived) organoids (TO) were established from the ERM obtained from the dental follicle of unerupted third molars [87]. Established human TO display a dental epithelial and stem cell phenotype similar to the ERM. Importantly, human TO are also able to mirror important biological functions of the ERM. Firstly, ERM are known to become proliferative and undergo EMT due to elevated EGF signals following infection, trauma, or orthodontic tooth movement [90]. This response to EGF is mimicked in vitro, when EGF-treated human TO become proliferative and undergo EMT. Secondly, human TO are amenable to both AB- and periodontal ligament-like differentiation in vitro. Developmentally, ERM are derived from HERS and have been ascribed to contribute to cementum and/or periodontal ligament repair and regeneration [91, 92]. Moreover, ERM are known to express enamel matrix proteins in vivo and in vitro cultured ERM were also shown to be able to acquire AB-like properties [87, 93, 94]. In addition, AB-like differentiation is also enhanced when co-cultured in the presence of dental pulp stem cells (DPSC) or when treated with TGFβ1 [87].

Mouse TO were established from early-postnatal (day 7) human-resembling molars and ever-growing incisors, and from 9-week-old adult mouse incisor [88, 89]. Both protocols rely on typical organoid medium components containing WNT pathway activation (WNT3A, RSPO1), BMP inhibition (NOGGIN), EGF (notably lacking from developed human TO medium), activin receptor-like kinase 5 (ALK5) inhibition (A83-01) as well as N-acetylcysteine, nicotinamide and fibroblast growth factor 10 (FGF10). The protocol developed by Kim et al. resulted in a less complex medium, also containing Notch signaling activator dibenzazepine, whereas the protocol established by our group also contained FGF2, FGF8, SHH, insulin growth factor 1 (IGF1) and cholera toxin (which activates adenylate cyclase and elevates intracellular cAMP levels).

In both cases, developed mouse TO contain abundant cytokeratin presence in the organoid cores, and closely mirror essential aspects of the dental epithelium (e.g. expression of dental epithelial stem cell markers SOX2 or TP63). Importantly, early-postnatal molar and incisor TO recapitulate tooth-type specific features (not yet evaluated for adult incisor TO). For instance, the transcription factor ISL LIM homeobox 1 (ISL1), which is essential for incisor dental epithelium but dispensable for molar development, is abundantly present in incisor-, but not molar TO [95]. Comparably to human TO, early postnatal molar and incisor TO also respond to exogenous EGF supplementation similarly to reported in vivo observations. Whereas EGF supplementation (also a key component for adult incisor TO) strongly improves incisor TO proliferation and induces precocious eruption of incisors when injected perinatally, no significant effects were reported for molar TO or eruption of molars [96, 97]. This faithful application is a powerful advantage compared to other currently available models and enables in vitro scrutiny of tooth-type specific biology.

Importantly, both studies reported unique protocols able to mirror distinct aspects of AB-like differentiation in vitro [88, 89]. Whereas suspension culture (which mirrors the loss of basement membrane between dental epithelium and dental mesenchyme during the transition from preAB to sAB) and SHH activation enable mirroring of the sAB phase (formation of crystals containing AMELX and AMBN), 3D culture in Matrigel with BMP and TGFβ activation predisposes toward the mAB stage (high ODAM/AMTN). Sequential combination of both approaches (i.e. a first differentiation phase in suspension, followed by a return to Matrigel) provides an exciting stepping stone toward full, in vitro recapitulation of amelogenesis in follow-up studies.

Taken together, these properties, together with the benefits of organoids over other available dental epithelial cell culture models, make tissue-derived TO a promising candidate for enamel and/or whole-tooth regeneration approaches as well as enamel disease modeling (Supplemental Appendix). Importantly, as previously mentioned, organoids or 3D spheroids can also be developed starting from iPSC/ESC instead of tissue-derived stem cells. Such models have recently also been successfully achieved for dental epithlium/AB-differentiated human iPSC, showing AB-like features and tooth-forming capability when combined with embryonic day 14.5 dental mesenchyme in vivo [50, 52, 53]. Recently, Alghadeer et al. successfully established 3D spheroids starting from iPSC-derived dental epithelial/AB-like cells [53]. Following a 16-day induction protocol, cells were transferred to ultra-low attachment plates and developed spheroids in suspension. Importantly, the developed ‘ameloblast spheroids’ displayed sAB-mirroring polarity, and were able to mimic polarity defects as seen in amelogenesis imperfecta. Joint implantation of ameloblast and DPSC spheroids in a mouse kidney capsule model resulted in secretion of enamel matrix proteins and formation of mineralized tissue. Because iPSC can be easily derived from skin fibroblasts, these models have the benefit of being developed without the need for tooth extraction (as opposed to human TO). Yet, as described above, the use of iPSCs is also associated with several disadvantages such as potential genetic alterations during reprogramming process, incomplete reprogramming and the epigenetic memory of source cells in addition to the risks associated with remnant undifferentiated iPSC (i.e. risk of tumor formation, off-target differentiation).

### Bioprinting Dental Epithelium

Bioprinting of 3D tissue constructs is a relatively new and rapidly growing technology that leverages conventional 3D printing of viable living cells embedded in an extracellular matrix (ECM)-like bioink to generate functional biomimetic tissues. Combination of tight spatial control with reproducible and high throughput production makes 3D bioprinting a powerful player in the field of regenerative and personalized medicine. Although beyond the scope of this review, and thoroughly covered elsewhere, crucial aspects for consideration when developing bioprinted tissue constructs are: (1) the cell source, (2) differentiation state of printed cells, (3) bioink and (4) printing strategy [98, 99]. Although numerous groups have achieved remarkable results in dentistry research (reviewed by Obregon et al. (2015) and Ostrovidov et al. (2023)), only limited studies have achieved bioprinting of dental epithelial tissues [100–103].

As native dental epithelial/AB cells are lost by apoptosis once enamel is formed, alternative cell sources must be used when 3D bioprinting dental epithelial/AB. Mohabatpour et al. (2022) combined HAT-7 cells, i.e. IDCEL, with a newly developed two-component bioink comprising alginate and carboxymethyl chitosan (water-soluble chitosan-derivative with anti-microbial and mucoadhesive properties) to bioprint scaffolds for enamel tissue regeneration [103]. Following 3D bioprinting in alginate- carboxymethyl chitosan, HAT-7 cells displayed high viability (> 80%), ALP expression and gene expression of AB markers (e.g. *Dentin sialophosphoprotein* (*DSPP)*, *AMBN*, *ENAM* and *KLK4*). Additionally, after 14 days, increased calcium and phosphorus content indicated initiation of mineralization. Taken together, these findings suggest suitability of such 3D scaffolds for differentiating dental epithelial cells toward AB-like cells and their potential for enamel bioengineering and repair/regeneration [103].

Tang et al. (2022) were able to 3D bioprint gelatin methacrylate (GelMA) constructs containing both primary rat dental papilla cells and HERS cells, derivates from the inner and outer enamel epithelium [102]. However, the main goal of this study was to promote essential epithelial-mesenchymal interactions during bone regeneration, rather than developing dental epithelial constructs. GelMA-encapsulated cells showed high viability (> 80%), proliferation, and migration. Notably, following tooth extraction, transplantation in the alveolar socket of Sprague-Dawley rats enhanced osteogenic differentiation (i.e. expression of osteogenic markers collagen type I, osteocalcin, Runt-related transcription factor 2 (RUNX2) and DSPP). *De novo* bone formation was enhanced in constructs containing both cell types.

Future studies aimed at applying 3D bioprinting for enamel repair/regeneration should commence from iPSC and/or organoid-derived dental epithelial material, given their reduced safety concerns. Indeed, bioprinting of advanced, highly mature iPSC/organoid-derived structures has been.

achieved. Both bioprinting of undifferentiated/uncommitted and differentiated (e.g. iPSC-derived cardiomyocytes, endothelial cells, oligodendrocyte precursors, neuronal precursors) have been successful to obtain mature tissue constructs [104–107]. Bioprinting can support generation of large-scale, highly reproducible organoids with enhanced maturation in a high throughput fashion, thereby overcoming some of the shortcomings of organoid technology [108]. Recently, bioprinting of centimeter-scale tissues was accomplished by Brassard et al. (2020), who applied a novel 3D bioprinting technique, the so-called bioprinting-assisted tissue emergence (BATE), using organoid-forming intestinal stem cells [109]. Notably, maturation and lumenization of bioprinted tissue constructs were achieved by co-deposition of intestinal mesenchymal cells. Together, high-throughput 3D bioprinting of reproducible, large-scale, mature tissues derived from iPSC and/or organoids holds tremendous potential to revolutionize drug discovery and regenerative/personalized medicine.

## Conclusion

As has become clear, the available tools for in vitro modeling of dental epithelium and AB have tremendously advanced in recent years. The onset of expandable organoid and iPSC models that are able to generate dental epithelium and AB-like cells, together with continuous advances in 3D bioprinting, provide exciting avenues for future research (i.e. tooth development, lineage specification, disease modeling) and have unlocked the door for developing strategies for biological enamel repair and regeneration (see Graphical Abstract). Importantly, we believe these differing approaches (e.g. iPSC versus organoids) are highly complementary and can cross-fertilize, where insights from one model can be applied to advance the other. Moreover, combination of these dental epithelium/AB models with the rapid and exciting developments in dental mesenchymal models (e.g. via co-culture, co-bioprinting), as well as breakthroughs in identifying and modulating tooth field-forming and -inhibiting signals (e.g. USAG-1, also known as SOSTDC1) will be the necessary next steps forward in the field [1, 110, 111].

## Data Availability

Not applicable.
